# 

**Published:** 1903-10-15

**Authors:** 


					﻿THE
DENTAL REGISTER
EDITED BY
NELVILLE S. HOFF, D. D.S.,
ANN ARBOR, MICH.
“Vol. LVH.-OCTOBER 15, 1903.	No. io.
PRICE, ONE DOLLAR A YEAR IN ADVANCE.
PUBLISHED MONTHLY BY
SAMUEL· A, CROCKER & CO.,
THE OHIO DENTAL AND SURGICAL DEPOT.
3» to 30 W. »tli ^t., Ciiicirinati, O.
Entered at the Postoffice at Cincinnati, 0., as second-class mail matter.
				

